# Relationships between the integrity and function of lumbar nerve roots as assessed by diffusion tensor imaging and neurophysiology

**DOI:** 10.1007/s00234-017-1869-0

**Published:** 2017-07-25

**Authors:** S. Y. Chiou, P. J. Hellyer, D. J. Sharp, R. D. Newbould, M. C. Patel, P. H. Strutton

**Affiliations:** 1 The Nick Davey Laboratory, Division of Surgery, Human Performance Group, Department of Surgery and Cancer, Faculty of Medicine, Imperial College London, London, UK; 2 Computational, Cognitive and Clinical Neuroimaging Laboratory, Division of Brain Sciences, Imperial College London, London, UK; 3 Department of Bioengineering, Imperial College London, London, UK; 4 Imanova, Ltd, London, UK; 5 Imaging Department, Imperial College Healthcare NHS Trust, Charing Cross Hospital, London, UK

**Keywords:** Diffusion tensor imaging, Nerve integrity, Spinal nerve roots, Transcranial magnetic stimulation, Electrical stimulation, Neurophysiology

## Abstract

**Purpose:**

Diffusion tensor imaging (DTI) has shown promise in the measurement of peripheral nerve integrity, although the optimal way to apply the technique for the study of lumbar spinal nerves is unclear. The aims of this study are to use an improved DTI acquisition to investigate lumbar nerve root integrity and correlate this with functional measures using neurophysiology.

**Methods:**

Twenty healthy volunteers underwent 3 T DTI of the L5/S1 area. Regions of interest were applied to L5 and S1 nerve roots, and DTI metrics (fractional anisotropy, mean, axial and radial diffusivity) were derived. Neurophysiological measures were obtained from muscles innervated by L5/S1 nerves; these included the slope of motor-evoked potential input-output curves, F-wave latency, maximal motor response, and central and peripheral motor conduction times.

**Results:**

DTI metrics were similar between the left and right sides and between vertebral levels. Conversely, significant differences in DTI measures were seen along the course of the nerves. Regression analyses revealed that DTI metrics of the L5 nerve correlated with neurophysiological measures from the muscle innervated by it.

**Conclusion:**

The current findings suggest that DTI has the potential to be used for assessing lumbar spinal nerve integrity and that parameters derived from DTI provide quantitative information which reflects their function.

## Introduction

Diffusion tensor imaging (DTI) can be used to characterise the microstructure of tissue in vivo [1]. It has been applied in the central nervous system (CNS), and studies have shown the clinical application of using DTI in the evaluation and diagnosis of a number of conditions including stroke, traumatic brain injury, and multiple sclerosis [2–5]. Recent work has demonstrated the feasibility of using DTI to measure the integrity of peripheral nerves [6–9], and a recent meta-analysis suggests that fractional anisotropy (FA) may be a useful measure of median nerve structure in patients with carpal tunnel syndrome (CTS) [10].

Compression of lower lumbar spinal nerves caused by herniation of the intervertebral disc is a common condition, with a prevalence of 10 to 25% in Europe [11]; the incidence peaks occur in the fifth decade. It can lead to severe pain in the legs (sciatica) and lower back as well as functional disability, which place enormous burden on health services [12, 13]. Spinal surgery is often performed to relieve leg pain if conservative treatment is not successful and pain persists into chronicity; the outcome of surgery, however, is highly variable [14]. Currently, the primary diagnostic indicator for the surgical decision-making process is spinal magnetic resonance imaging (MRI), but discrepancies between symptoms and MRI findings are frequently observed [15, 16]. Although conventional MRI can be used to determine the location of nerve compression, it does not permit prediction of the effects of treatment, as the extent of nerve damage is difficult to estimate.

DTI has the potential to complement standard MRI, as it provides a quantitative measure of tissue microstructure and nerve integrity [17]. However, DTI of the lumbar spine is challenging, given the relatively small cross-sectional area of the lumbar nerve roots, the presence of susceptibility artefacts around the spinal cord due to tissue-bone interfaces and the co-localisation of large stores of body fat [18, 19]. Despite these challenges, initial work using DTI and tractography to visualise and evaluate these nerves in healthy subjects and in patients with lumbar nerve compression has shown promising results. Lower fractional anisotropy (FA) and higher mean diffusivity (MD) or apparent diffusion coefficient (ADC) have been observed in compressed nerve roots, compared to both contralateral uncompressed nerve roots and equivalent nerve roots in healthy subjects [20–23].

The function of nerve roots can be assessed using neurophysiology, and this, combined with DTI, provides an opportunity to test the degree to which alteration in nerve integrity relates to function. Work in the central nervous system shows correlations between diffusion metrics and neurophysiological measures. For example, corpus callosum DTI parameters correlate with interhemispheric inhibition measured by paired-pulse transcranial magnetic stimulation (TMS) [24], suggesting that structural abnormalities demonstrated by DTI have a functional impact. The function of lumbar spinal nerves is routinely assessed using neurophysiological testing (e.g. nerve conduction studies) and is altered in patients with radiculopathy [25–27]. However, whether measures of nerve microstructure assessed using DTI reflect function in these nerves remains to be established.

In this study, we first examine whether the diffusion metrics derived from our optimised DTI acquisition are comparable between sides and across nerves. Fibre-tracking was also applied to test whether the tractography fibre bundle can be tracked and matched to the lumbar nerve roots on the T2-weighted MRI images. Next, we investigated the relationships between lumbar spinal nerve integrity (measured using DTI) and function (using neurophysiological measurements) of the nerve roots (and muscles innervated by) L5 and S1 in healthy subjects; lumbar disc herniation most frequently compresses these nerve roots. Specifically, we test the hypothesis that measures of microstructure such as FA, MD, axial and radial diffusivity (AD and RD) correlate with neurophysiological measurements reflecting the function of the peripheral nervous system (e.g. F-wave latency).

## Materials and methods

### Participants

With ethical approval and written informed consent, 20 healthy adults (12 male; mean ± SD age, 33.3 ± 8.91 years; height, 1.71 ± 0.10 m) were recruited from students and staff at the authors’ institution. Participants were excluded if they had a history of musculoskeletal abnormalities of the back musculature, axial skeleton or lower limbs (e.g. low back pain, radiculopathy). Further, exclusion criteria related to the use of TMS and of MRI (i.e. metal implants, cardiac pacemaker, history of epilepsy or fits, previous brain injury, neurosurgery, neurological disorders, psychological disorders, actively taking antidepressants or other neuromodulatory drugs).

### MRI data acquisition

All MRI data were collected using a 3T Siemens Verio clinical MRI scanner (Siemens Healthcare, Erlangen Germany). Subjects were imaged supine using an 11 cm local loop coil centred over the intervertebral disc between L5 and S1 in combination with two elements of the phased-array spine coil to maximise the signal-to-noise ratio in the lumbar roots to further improve the diffusion imaging. Correct coil positioning was verified by initial localizer scans. Structural imaging was reviewed by a consultant radiologist to confirm no evidence of lumbar nerve compression; this included sagittal T1-weighted (T1w) and T2-weighted (T2w) turbo spin echo (TSE), coronal T2w TSE, as well as a multislab T2w TSE angled axially to the L3-L4, L4-L5, and L5-S1 vertebral discs. Diffusion-weighted images (DWI) were acquired with *b* = 800 s/mm^2^ using a twice-refocused diffusion preparation, an inverted slice select gradient on the refocusing pulses for improved fat saturation [28], and a 2D EPI readout. Forty 2.5 mm thick adjacent slices of a 100 × 256 mm field of view (FOV) were collected with TE = 92 ms, TR = 9 s and 50 × 128 resolution with readout bandwidth of 1562 Hz per pixel, giving a resolution of 2.0 × 2.0 × 2.5 mm. The effectiveness of using a reduced phase-encoding field of view (FoV) to reduce susceptibility-induced distortions in lumbar nerve imaging has been demonstrated [19, 29]. Saturation bands were placed superiorly and inferiorly to the imaging slab to reduce flow and off-resonance excitation artefacts. Sixty-four non-collinear directions interspersed with a *b* = 0 measurement after every 16 directions were collected resulting in 68 acquisitions in 10 m:21 s.

### Neurophysiological measurements

#### Recording

Electromyographic (EMG) recordings were obtained bilaterally from the target muscles, tibialis anterior (TA) and soleus (SOL). Pairs of Ag/AgCl electrodes (self-adhesive, 2 cm diameter, CareFusion, UK) were positioned parallel to the muscle fibre orientation. A ground electrode was placed over the left lateral malleolus. For TA, electrodes were positioned at one third way down a line between the head of the fibula and the superior aspect of the medial malleolus; for SOL, two-thirds way down a line between the medial condyle of the femur and the medial malleolus. Participants were additionally asked to contract the target muscles by ankle dorsiflexion or plantarflexion to confirm that the electrodes were located over the most prominent muscle bulk. EMG data were filtered (10–1000 Hz), amplified (1000×; Iso-DAM, World Precision Instruments, UK) and sampled at 2 kHz using a Power 1401 data acquisition system and Signal v5 software (Cambridge Electronic Design [CED], UK) connected to a computer for subsequent offline analysis.

#### TMS

TMS was delivered to the motor cortex using a Magstim 200^2^ mono-phasic stimulator (The Magstim Company Ltd., UK) connected to a figure-of-eight coil (wing outer diameter 10 cm), positioned over the approximate location of primary motor cortex at a site which elicited a maximal motor-evoked potential (MEP) in the contralateral target muscle.

#### Experimental parameters

Measurements were conducted, while participants were seated in an armchair with torso supported by the backrest and feet strapped securely on a wood plate on the floor. Three brief (~2 s) maximum voluntary contractions (MVC), with at least 10 s rest between contractions, were recorded from each target muscle; strong verbal encouragement was provided throughout. The mean rectified EMG over 500 ms during each of the three MVCs was calculated and averaged, and 10% of this value was displayed continuously on a screen as visual feedback for participants during all TMS measurements.

#### Corticospinal excitability

Measurements were performed on each target muscle separately and while participants maintained contraction levels at 10% MVC of the target muscle. Active motor threshold (AMT) was established for each target muscle, which was defined as the lowest intensity of TMS that evoked visible MEPs in at least three of six consecutive trials. Motor-evoked potentials (MEPs) were evoked by TMS, and an input-out relationship of MEP amplitude to stimulus intensity (IO curve) was constructed. Stimulus intensities started at 10% below the AMT and were increased in 10% steps of AMT until the intensity reached to the maximal device output. Intensities were randomised and six MEPs at each intensity were recorded. TMS pulses were given every 8 s with several periods of rest given to participants between trials to avoid muscle fatigue.

#### M-wave and F-waves

A maximal motor response (*M*_max_) and F-waves were measured with the use of supramaximal stimuli via a cathode to the common peroneal nerve around the fibular head (for TA) or the tibial nerve in the popliteal fossa (for SOL) (Digitimer DS7, Digitimer UK, 500-μs pulse duration). The anode was placed over the patella on the stimulated side. Five *M*_max_ at the same intensity were recorded; an intensity of 120% of the intensity used to elicit *M*_max_ was delivered to the nerve at 1 Hz until 20 F-waves were recorded.

### Data analysis

#### DTI

**Fig. 1:**
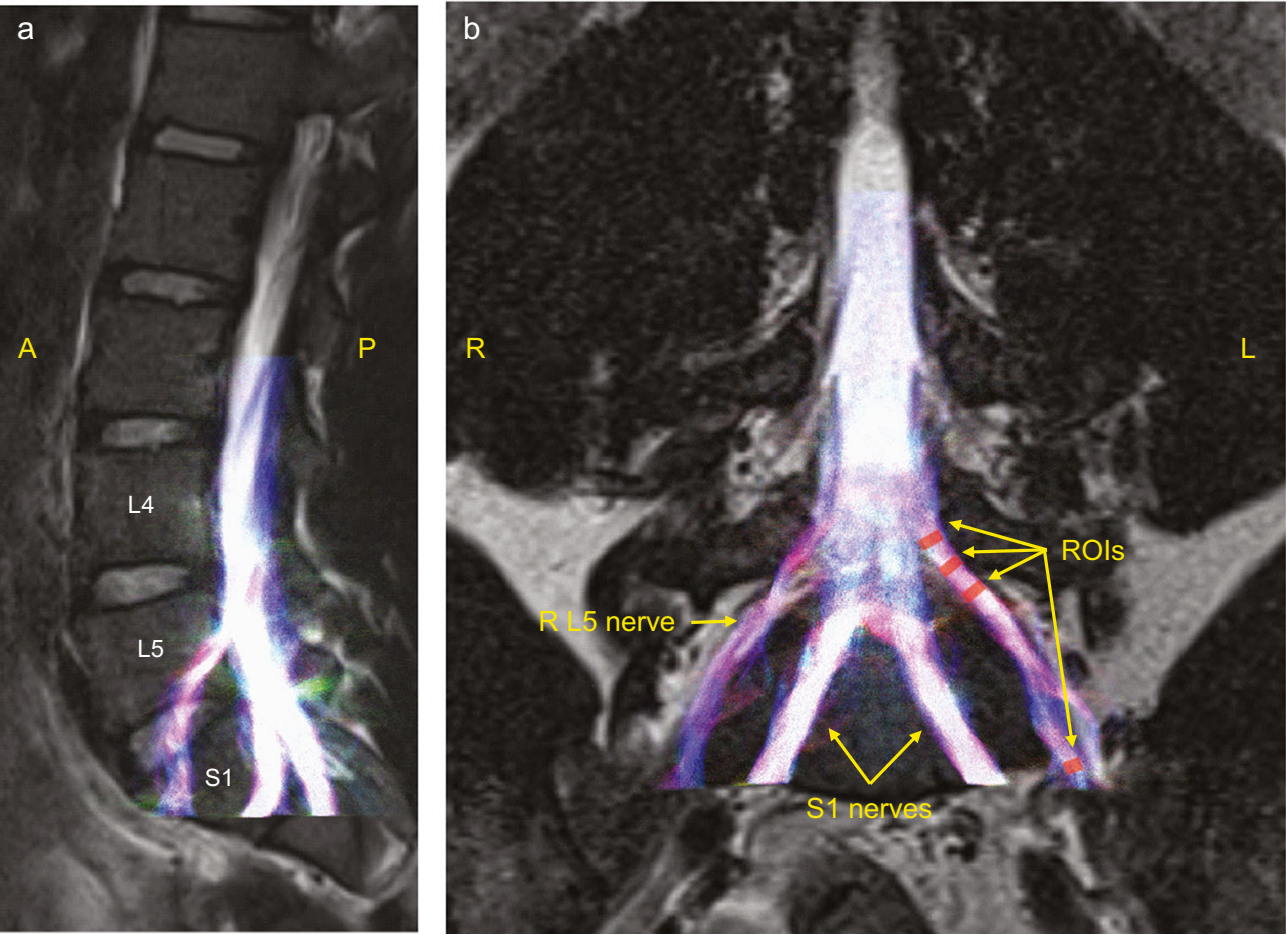
Overlaid diffusion tensor tractography of L5 and S1 nerves on the T2-weighted sagittal (**a**) and coronal (**b**) images of the lumbar spine from a representative subject. Tractography shows the anatomical orientation of L5 and S1 nerves. The mean orientation of the streamlines is indicated in *colours*: left-right (*red*), anterior-posterior (*green*) and inferior-superior (*blue*). Regions of interests as described in the “Methods” are illustrated for the left L5 nerve. The regions of interest (ROIs) from proximal to distal for the L5 nerve are at ~14, ~20 and ~28 mm inferior to the disc and at the distal end of the nerve. *R* right, *L* left, *A* anterior, *P* posterior

Post-processing of diffusion-weighted images (DWI) and fitting of the diffusion tensor were performed using the FSL Diffusion Toolbox (FDT) (FSL, http://fsl.fmrib.ox.ac.uk/fsl) v.5.0.6 (Oxford, UK). The diffusion tensor was estimated using the FSP Diffusion Toolkit [30, 31]. Fractional anisotropy (FA), mean diffusivity (MD) and axial diffusivity (AD) maps were generated using FDT as well as voxel-wise estimates for each of the eigenvalues (*λ*1, *λ*2 and *λ*3) representing the magnitude of diffusion in the three principal directions. Radial (RD) diffusivity images were then derived from the eigenvalues (RD = *λ*2 + *λ*3/2). In order to evaluate these measures along the course of each of the L5 and S1 nerves on both the left and right side, a region of interest (ROI) approach was used. ROIs were manually drawn with reference to the axial view of *b* = 0 image from the diffusion acquisition overlaid onto the co-acquired axial T2-weighted image (see Fig. 1). Three binary ROIs were manually traced onto the image using FSLview (FSL, http://fsl.fmrib.ox.ac.uk/fsl) on both left and right L5 and S1 nerves below the disc ~14, ~20 and ~28 mm distal to the centre of the disc. A fourth ROI was placed over the most distal visible portion of the nerve visible within the field of view. Due to the close proximity of the L5 nerve to the central spinal canal, it was difficult to place with any degree of accuracy any ROIs above the level of the disc. However, for the S1 nerves, where clear differentiation could be made between nerve above the level of the disc and the spinal canal, an additional ROI was placed immediately above (within 2.5 mm of) the disc. Each ROI was drawn to cover the entire visible signal on the *b* = 0 image which was clearly differentiable as nerve, resulting in ROIs of between 40 and 60 mm^3^ (with a slice thickness of 2.5 mm, this equates to 16 and 24 mm^2^, respectively). This size of ROI reduces the partial volume effect; the neutral-position cross-sectional area of nerve roots in the lower lumbar region has been shown in a cadaveric study to be 34.48 ± 11.25 mm^2^ [32]. This manual ROI drawing generated a total of eight binary ROI masks. For each of the FA, MD, AD and RD images, ROI diffusion values were sampled from over-lapping voxels between each of the masks and the metric of interest. The average of each metric within each mask was subsequently taken forward into further analysis.

#### Fibre tracking

For visualisation, basic fibre tracking analysis was performed at the level of each of the L5 and S1 spinal nerves. Fibre orientation distribution (FOD) analysis and fibre tracking were performed on the DWI data using MRtrix (https://github.com/MRtrix3/mrtrix3). Voxelwise FODs were estimated using constrained spherical deconvolution [33], with an lmax of 6. Probabilistic tractography was performed the iFOD2 algorithm [33], with a step size of half the DWI voxel size, a turning angle threshold of 90° and an streamline termination threshold of FOD < 0.1. Tracks were seeded from the spinal canal superior to the L5 and S1 nerves, and terminated on entering the most distal ROI in each of the bilateral L5 and S1 nerves (see above). Streamlines were seeded at random from the seed until 10,000 streamlines successfully traversed from the seed to each of the target ROIs.

#### EMG

The mean MEP amplitude per stimulus intensity was calculated and normalised to the *M*_max_ for each muscle; this was defined as MEPmax. The mean MEP amplitudes between 110 and 170%AMT and between 110 and 140%AMT were used to calculate slopes for the TA and SOL, respectively; the slopes (IO_slope_) were defined as the steepness of the linear regression line for the given data points [34]. Mean pre-stimulus EMG was calculated in a 100-ms window from the rectified EMG traces for the TA and SOL at each intensity. The average rectified EMG trace from the trials in which 120% AMT was delivered was used to derive the MEP latency for each muscle. The amplitude and latency of averaged *M*_max_ were measured, and the minimum latency of F-waves was identified from the recorded 20 F-waves. Central motor conduction time (CMCT) and peripheral motor conduction time (PMCT) were calculated using the following equations:

$$ CMCT(ms)=MEP\  latency-\left(\frac{M\mathit{\max}\  latency+\mathit{\min}.F\  wave\  latency-1}{2}\right) PMCT(ms)=\frac{M\mathit{\max}\  latency+\mathit{\min}.F\  wave\  latency-1}{2} $$

### Statistical analysis

Data were analysed using SPSS 21 (IBM Corp, Armonk, NY). To fully describe the relationship between nerve “Level” (L5 vs. S1), “Side” (left vs. right) and “ROI” (four ROIs distal to the disc) in healthy controls, repeated measures ANOVA with post hoc *t* tests were used. The same analysis was carried out to examine the effect of “Intensity” and “Side” on IO curves and pre-stimulus EMG for the TA and SOL. Paired *t* tests were used to examine side differences on IO_slope_, *M*_max_, minimum F-wave latency, CMCT and PMCT. To establish a relationship between DTI parameters and neurophysiological measurements, regression analysis was employed for DTI metrics of L5 and S1 separately with the neurophysiological measurements obtained from their corresponding muscles. The DTI parameters from the ROI closest to the level of the disc were used in this analysis. Neurophysiological parameters were IO_slope_, *M*_max_, minimum F-wave latency, CMCT and PMCT. Age, gender and body height were included as covariates of no interest. Statistical significance was set at *p* < 0.05, and Bonferroni correction was applied to adjust for multiple comparisons. Further, Pearson correlation coefficient analyses were performed on DTI metrics and TMS parameters to examine any correlation between L5 and S1 nerves and between TA and SOL. Data are presented as mean ± SD in the text and as mean ± SEM in the figures.

## Results

### DTI and fibre tracking of lumbar nerve roots

The lumbar nerve roots were well visualised in the diffusion-weighted acquisitions, and fibre tracking was successful in all 20 subjects. A representative dataset is shown in Fig. 1, demonstrating the excellent co-localization between the geometrically accurate TSE and the optimised diffusion-weighted EPI that is well-known for geometric distortion. Table 1 shows DTI parameters. FA, MD, AD and RD values were very similar between left and right sides, showing no significant side differences. Further, there were no significant differences between vertebral levels (L5 and S1) in these measures.

**Table 1:** Diffusion tensor imaging (DTI) metrics and results of statistical analyses

DTI metric	Level (L5, S1)	Side (left, right)	ROI (~14, ~20, ~28 mm, distal)
FA	*F*_1,14_ = 0.39; *p* = 0.54	*F*_1,14_ = 0.15; *p* = 0.71	*F*_3,42_ = 9.42; *p* < 0.001
MD	*F*_1,14_ = 1.69; *p* = 0.21	*F*_1,14_ = 0.04; *p* = 0.85	*F*_3,42_ = 15.27; *p* < 0.001
AD	*F*_1,14_ = 1.05; *p* = 0.32	*F*_1,14_ = 0.008; *p* = 0.93	*F*_3,42_ = 14.58; *p* < 0.001
RD	*F*_1,14_ = 1.86; *p* = 0.20	*F*_1,14_ = 0.05; *p* = 0.82	*F*_3,42_ = 15.15; *p* < 0.001

*FA* fractional anisotropy, *MD* mean diffusivity, *AD* axial diffusivity, *RD* radial diffusivity, *ROI* region of interest

### Changes in DTI metrics along the course of the nerve

**Fig. 2:**
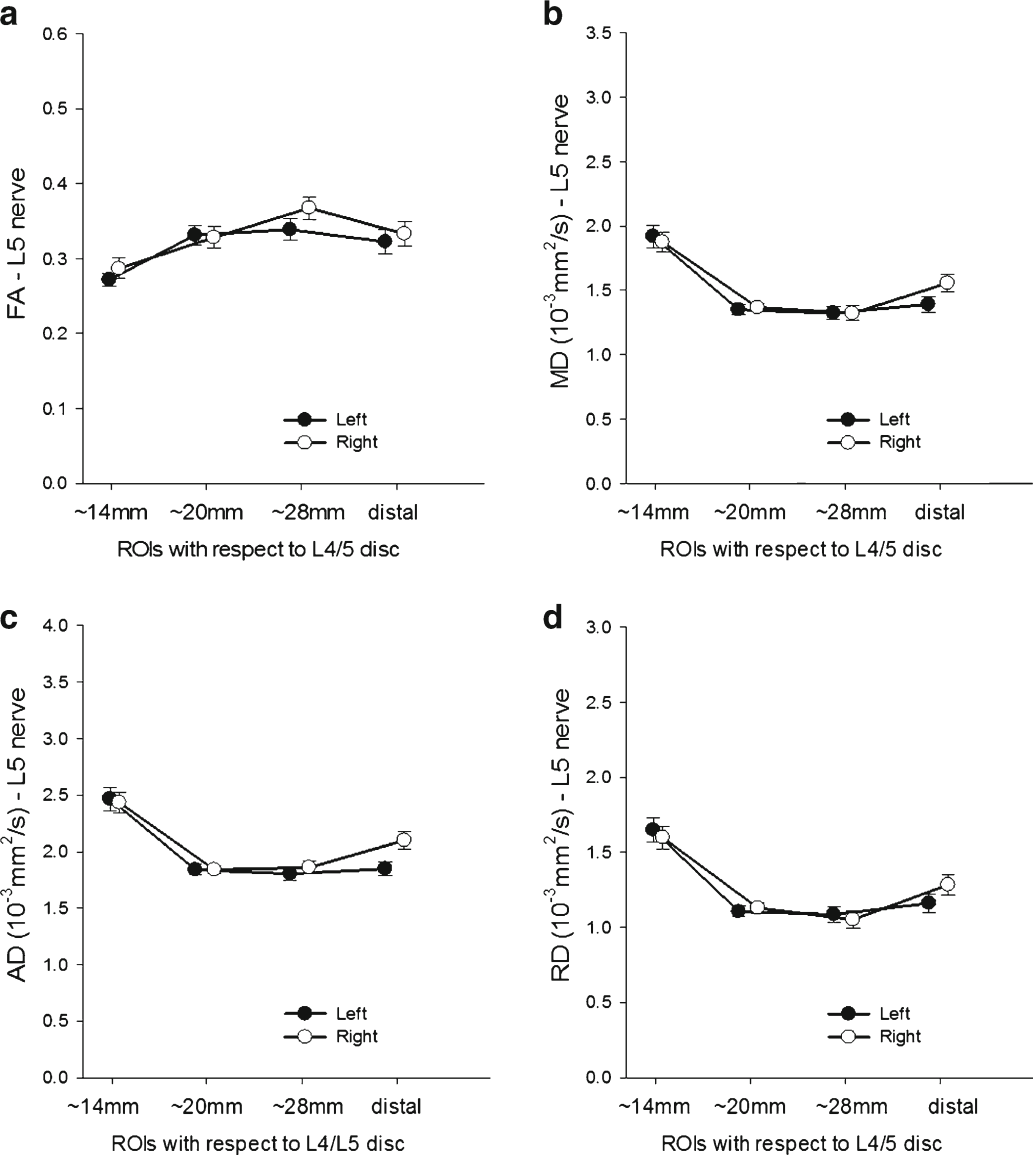

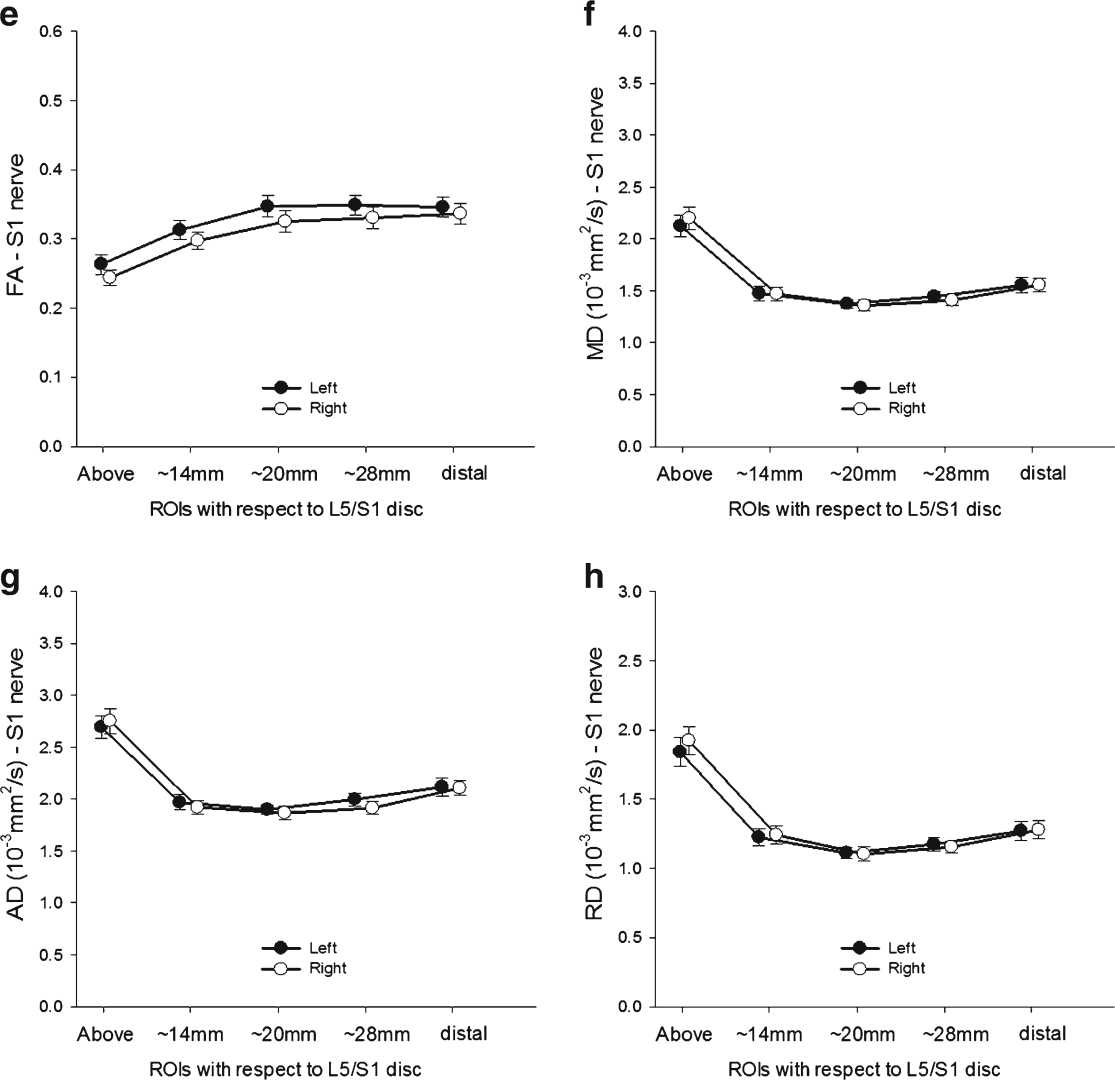
Diffusion tensor imaging (DTI) metrics along the course of the left and right L5 (**a**–**d**) and S1 (**e**–**h**) nerves. Data are presented as mean ± standard error

The diffusion metrics were significantly different along the course of the nerve with FA increasing in proximal-to-distal, and MD, AD and RD decreasing (Fig. 2). Post hoc paired *t* tests showed that the FA obtained from the most proximal portions of the nerves was significantly lower than that from the others (*p* < 0.05). The paired *t* tests showed that the MD, AD and RD obtained from the most proximal portions of the nerves were higher than that from the other ROIs (*p* < 0.05).

### Within-subject variation in neurophysiological measurements

**Fig. 3:**
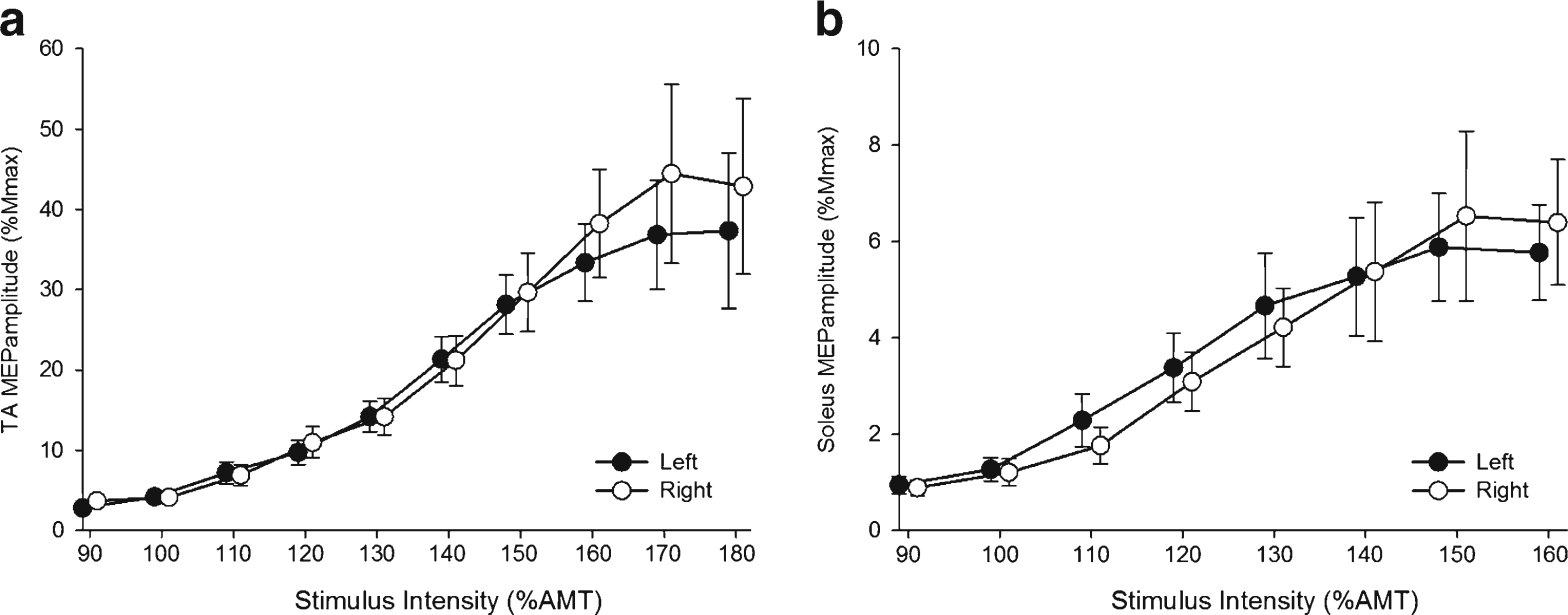
Input-output curves of motor-evoked potentials (MEP) from tibialis anterior (*TA*; **a**) and soleus (**b**) that display mean MEP amplitude on the *y*-axis against the stimulus intensity on the *x*-axis. The MEP amplitude was normalised to *M*_max_, and the stimulus intensity was expressed as the percentage of active motor threshold (*AMT*). Data are presented as mean ± standard error

The normalised amplitudes of MEPs in both TA and SOL increased with increasing stimulus intensity (both *p* < 0.05), but there were no laterality differences (Fig. 3). Additionally, there were no laterality differences in the slope of input-output curves of MEP amplitude against stimulus intensity (IO_slope_), maximal motor response (*M*_max_), minimum F-wave latency, and central and peripheral motor conduction time (CMCT and PMCT) in either TA or SOL (Table 2).

**Table 2:** Neurophysiological parameters and results of statistical analyses

Parameter	Tibialis anterior			Soleus		
	Left	Right	*p* value	Left	Right	*p* value
IO curve slope (MEP amplitude/%AMT)	1.28 ± 0.95	1.46 ± 1.12	0.50	0.26 ± 0.30	0.26 ± 0.24	0.94
*M*_max_ (mV)	4.97 ± 2.02	5.60 ± 2.32	0.19	12.62 ± 8.58	11.81 ± 7.19	0.62
Minimum F-wave latency (ms)	34.40 ± 3.77	35.21 ± 3.47	0.11	32.01 ± 3.21	32.21 ± 2.20	0.68
CMCT (ms)	12.74 ± 2.13	12.18 ± 2.72	0.13	13.85 ± 3.21	14.00 ± 3.03	0.84
PMCT (ms)	18.04 ± 1.91	18.33 ± 1.80	0.28	17.32 ± 1.20	17.45 ± 1.22	0.59

Data are presented as mean ± standard deviation

*IO curve* input-output curve, *MSO* maximal stimulate output, *M*_*max*_ maximal motor response, *CMCT* central motor conduction time, *PMCT* peripheral motor conduction time

### L5 DTI metrics correlate with maximal motor response and latency

**Fig. 4:**
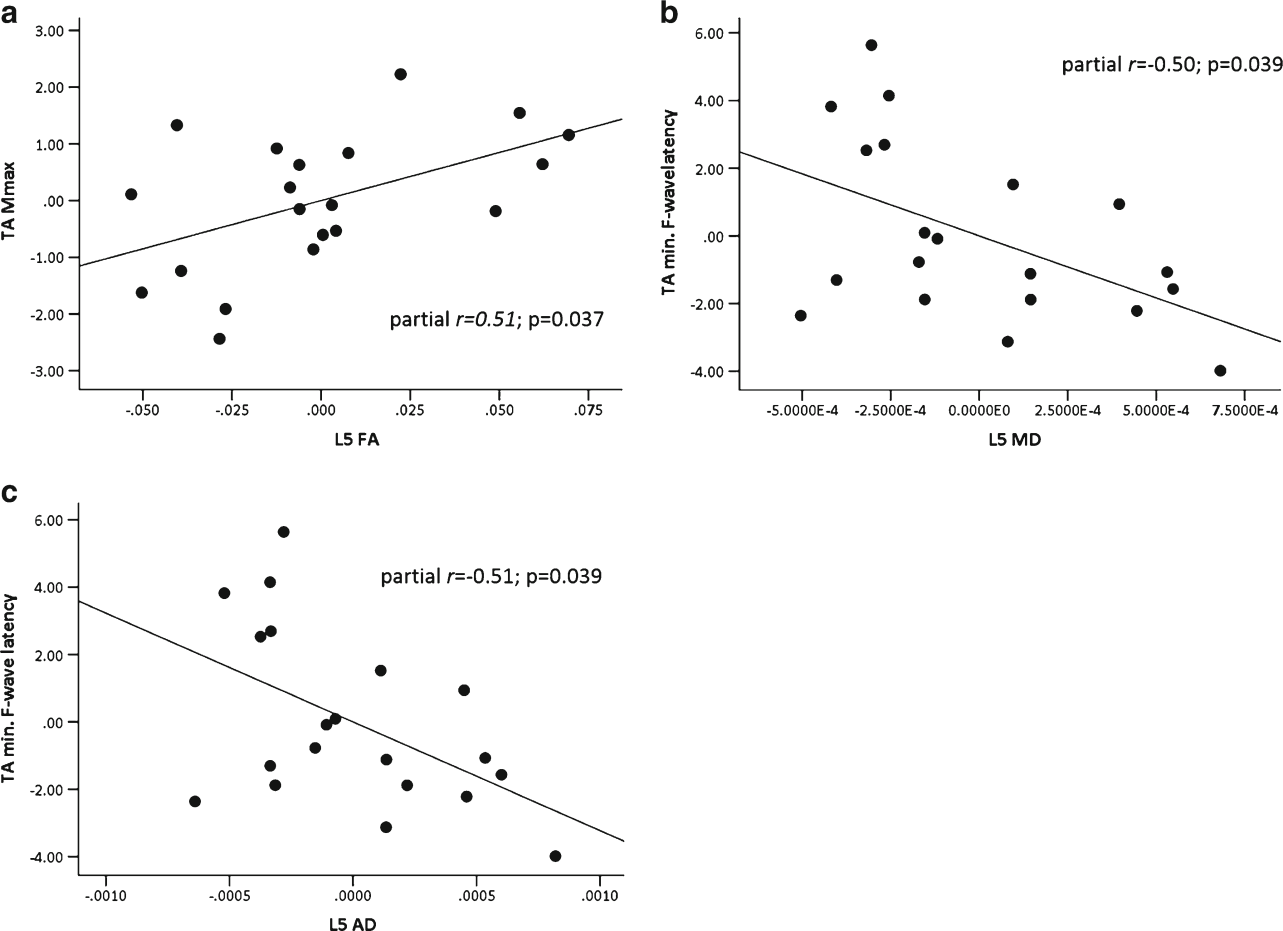
Relationships between DTI metrics of L5 and neurophysiological measurements obtained from the tibialis anterior (*TA*). Partial regression plots showing **a** fractional anisotropy (*FA*) correlated with the *M*_max_, **b** mean diffusivity and **c** axial diffusivity both correlated with minimum F-wave latency. The *x*- and *y*-scales of the partial correlation plots represent the adjusted values of DTI metrics and neurophysiological measurements after including age, gender and height as covariants

Since there were no laterality differences in DTI metrics or neurophysiological measurements, data from left and right sides were averaged prior to regression analyses. Regression analysis showed that the DTI metrics correlated with the function of the peripheral nerves. Specifically, L5 FA was correlated with the amplitude of maximal motor response (*M*_max_) obtained from TA, the effector muscle of L5 (overall *F*_4,15_ = 8.72; *p* = 0.001; partial correlation *r* = 0.51, *p* = 0.037; Fig. 4a). In addition, both MD (overall *F*_4,15_ = 7.63; *p* = 0.001; partial correlation *r* = −0.5, *p* = 0.039; Fig. 4b) and AD (overall *F*_4,15_ = 7.64; *p* = 0.001; partial correlation *r* = −0.51, *p* = 0.039; Fig. 4c) of L5 were correlated with the minimum F-wave latency obtained from TA. There were no correlations between S1 DTI metrics and neurophysiological measurements from SOL.

## Discussion

In the present study, DTI parameters of lumbar spinal nerves and their relationships with neurophysiological measures were investigated in healthy subjects. The DTI metrics derived from our diffusion imaging were consistent across left and right sides of the nerves at a given level, between L5 and S1 levels at a particular location, and across individuals even though the number of subject was limited. In addition, tractography of L5 and S1 nerves was successfully obtained and well localised to the lumbar nerves on the T2-weighted MRI. These suggest that our optimised DTI acquisition can provide stable DTI measures; a study to determine if they detect abnormalities in clinical populations is currently in progress. In addition, our results suggest that the integrity of the lumbar spinal nerves was different along the course of the nerve. FA increased from proximal to distal, while MD, AD and RD decreased. Further, we show that the MD and AD of the L5 nerve correlated with the minimum F-wave latency measured from the TA muscle, while the FA correlated with the amplitude of maximum motor response of the TA. The current findings provide a preliminary set of normative values of diffusion metrics for L5 and S1 nerves and show that the metrics, at least at L5, reflect the function of the nerves.

The feasibility of using diffusion MRI to derive DTI parameters from lumbar spinal nerves has been reported previously [20–22, 35, 36]. The majority of those studies measured DTI values at a single location of the nerves and reported no differences in FA, MD and ADC between left and right sides of the nerves as well as between L5 and S1 levels in healthy subjects. Only one of these studies [35] used multiple ROIs to characterise FA and ADC values along the course of the L5 and S1 nerve roots. Their findings, from a small sample of six healthy subjects, showed that FA increased distally from the junction of the dura mater while the ADC decreased. DTI acquisitions in the lumbar spine are compromised by poor magnetic field homogeneity. This results in spatially varying chemically selective (fat) saturation and pronounced geometric image distortion. Prior studies have relied on full field of view acquisitions. Reduction in the phase-encoding FOV linearly decreases the geometric distortion in that direction. The readout direction uses a high readout bandwidth that is often two orders of magnitude higher than that in the phase-encoding direction. A reduced phase-encoding FOV is essential for lumbar DTI [20]. Robust fat saturation was achieved in this study by using an inverted slice selection gradient on the refocusing pulses, not used in any of the previous studies. The majority of prior studies used chemically selective saturation of the fat signal, which fails in the presence of magnetic field inhomogeneities as expected here. Some prior studies utilised fat-selective inversion to improve fat saturation [20, 22]; however, that technique is sensitive to both field and RF inhomogeneities. Our work substantiates and extends previous work by using a multiple ROI approach on the L5 and S1 nerve roots in a preliminary cohort of 20 healthy subjects and, in addition to deriving FA and MD, also calculated AD and RD, which reflect perpendicular and parallel diffusivity, respectively. Our results show increases in FA in a proximal to distal direction and decreases in MD, AD and RD with little variation between sides and levels (L5, S1). This suggests that our imaging approach might be able to detect subtle changes occurring in L5 and S1 nerves which could be useful in monitoring surgical recovery in patient populations and warrants further investigation in a larger cohort.

Changes in DTI metrics along a proximo-distal course have been reported previously for the median nerve in the forearm, wrist and hand in healthy subjects, with a minimum FA in the centre of the carpal tunnel and maximum MD and RD values at the same location [37]. The authors suggested that the reduction in FA in the median nerve as it crosses the carpal tunnel reflects a possible entrapment site. The lumbar nerve roots are frequently compressed by herniated discs or narrowing foramina, and studies have shown that FA and ADC or MD are altered, with decreases in FA and/or increases in ADC or MD, in the compressed roots in comparison with the uncompressed roots [20–23, 38, 39]. Lower FA and/or higher ADC or MD in the compressed nerve roots suggest changes in diffusion direction and an increase in water diffusion which might indicate altered microstructure occurring in the compressed nerve. These findings show clinical utility of DTI to detect nerve impingement caused by a number of spinal pathologies, such as intervertebral disc herniation, degenerative disc disease, foraminal stenosis and spondylolisthesis.

It is not well established which features of nerve structure are revealed by DTI measures; functional assessment, e.g. using neurophysiological testing, is therefore required. Our results show that MD and AD in L5 root correlated with the minimum F-wave latency, reflecting conduction time along the nerve [40], from stimulation of the common fibular nerve, while the FA in the L5 root correlated with the maximum motor response (*M*_max_). The increase in MD (often called ADC) has been shown to be associated with increased tissue water levels caused by inflammation in multiple sclerosis [41, 42] or oedema in cerebral cancer [43]; however, correlations with neurophysiological function in these clinical populations are unclear. Studies on the median nerve have shown correlations between MD and nerve conduction velocities in patients with carpal tunnel syndrome, substantiating our current findings. Further, our results of a correlation between FA and *M*_max_ are consistent with some previous findings of the extent of axonal injury in animal models of nerve injury and in humans with neuropathy [44–47] but in contrast to a study showing FA correlated with distal motor latency, thought to reflect myelin integrity [37]. Other discrepancies between DTI measures and function are evident from previous studies. Correlations between AD and nerve conduction velocities of the median nerve have been reported in patients with carpal tunnel syndrome [10, 48], suggesting that AD relates to integrity of myelin. On the contrary, a recent study in healthy subjects showed that AD of the median nerve correlated with compound motor action potentials (CMAP) amplitudes, presumed to relate to axon integrity [37]. Several possible explanations might explain these discrepancies. Firstly, it is possible that the relationships between DTI and neurophysiological measures are more complex, since single measures derived from DTI might not accurately reflect only one aspect of nerve structure (e.g. myelin or axonal integrity). Further, in patient populations, there will be structural changes due to pathology which are revealed as functional deficits. In healthy subjects, there is no such pathology, and the relationships found in patients might not be evident. Secondly, clinical neurophysiological findings do not always match symptoms. This again suggests that the relationships between structure and function of the nerves might be more complex and multi-faceted.

Evidence has shown that L5 and S1 nerve roots respond to neurophysiological testing differently. Previous work on the sensitivity of neurophysiological testing for each level of lumbar nerve root lesion found that the percentage of positive pathological results on EMG and in F-wave latencies were higher in L5 nerve root lesions than in S1 nerve root lesions [49]. This may also explain the correlation between neurophysiology, and DTI was not evident for the S1 nerve root but was found for the L5 nerve root. Alternatively, the discrepancy in correlations could be explained by the lack of variability in our healthy subject data; the DTI data were consistent and the neurophysiological data were within normal ranges [50, 51]. It is therefore possible that associations might be revealed under pathological conditions, where changes in both neurophysiological and structural parameters are likely; a further study of the relationships between DTI metrics and neurophysiological measures in patients with compressed lumbar nerve roots is currently in progress.

In conclusion, the current findings provide a preliminary set of normative values of diffusion metrics for L5 and S1 nerve roots which suggests the potential for this DTI technique to be used in assessing lumbar spinal nerves in clinical settings as the values from the healthy subjects were highly consistent across the nerves of both sides as well as across levels. The relationships between DTI parameters and neurophysiological measures demonstrate the utility of using DTI metrics as a measure of nerve integrity and function. The current findings have clinical implications; the parameters derived from DTI provide quantitative information on lumbar spinal nerves which reflects their function. Whether these parameters can predict the likelihood of recovery of function following therapeutic interventions (e.g. surgery) in pathology remains to be established.
